# Supplementary material for: Stress Effect of Food Matrices on Viability of Probiotic Cells during Model Digestion
Source: Microorganisms. 2021 Jul 30;9(8):1625. doi: 10.3390/microorganisms9081625 (PMC8401621; doi:10.3390/microorganisms9081625)
Supplement: Supplementary file 1 [file microorganisms-09-01625-s001.zip › microorganisms-1305429-supplementary.pdf]

microorganisms

Supplementry File

Article

Stress Effect of Food Matrices on Viability of Probiotic Cells during Model Digestion

Petra Matouskova [1], Julie Hoova [1], Petr Rysavka [2] and Ivana Marova [1,*]

[1] Faculty of Chemistry, Brno University of Technology, Purkynova 118, 61200 Brno, Czech Republic; matouskova@fch.vut.cz (P.M.); xchoova@fch.vut.cz (J.H.)
[2] Pharmaceutical Biotechnology, Ltd., Slezska 949/32, 12000 Prague, Czech Republic; rysavka.petr@seznam.cz
* Correspondence: marova@fch.vut.cz; Tel.: +420-541149419

List of Figures:

Figure S1: Bacteria *Lactobacilus acidophilus, Bifidobacterium breve* and commercial probiotic mixture cultivated for 48 hours in MRS agar

Figure S2. Viability of probiotics analyzed by flow cytometry – results of analysis of commercial probiotic mixture incubated in water medium.

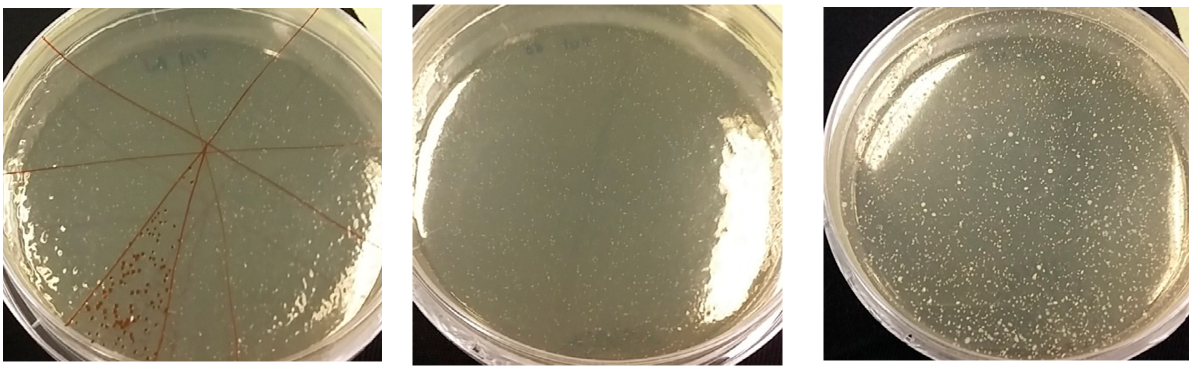

Lactobacilus acidophilus *Bifidobacterium breve* Commercial probiotic mixture

Figure S1: Bacteria *Lactobacilus acidophilus*, *Bifidobacterium breve* and commercial probiotic mixture cultivated for 48 hours in MRS agar

Note: During analysis of viability by cultivation method, cells in model and real samples were diluted to approx. 10^4 cells, placed on Petri dish and over-layered by agar medium to reduce oxygen presence.

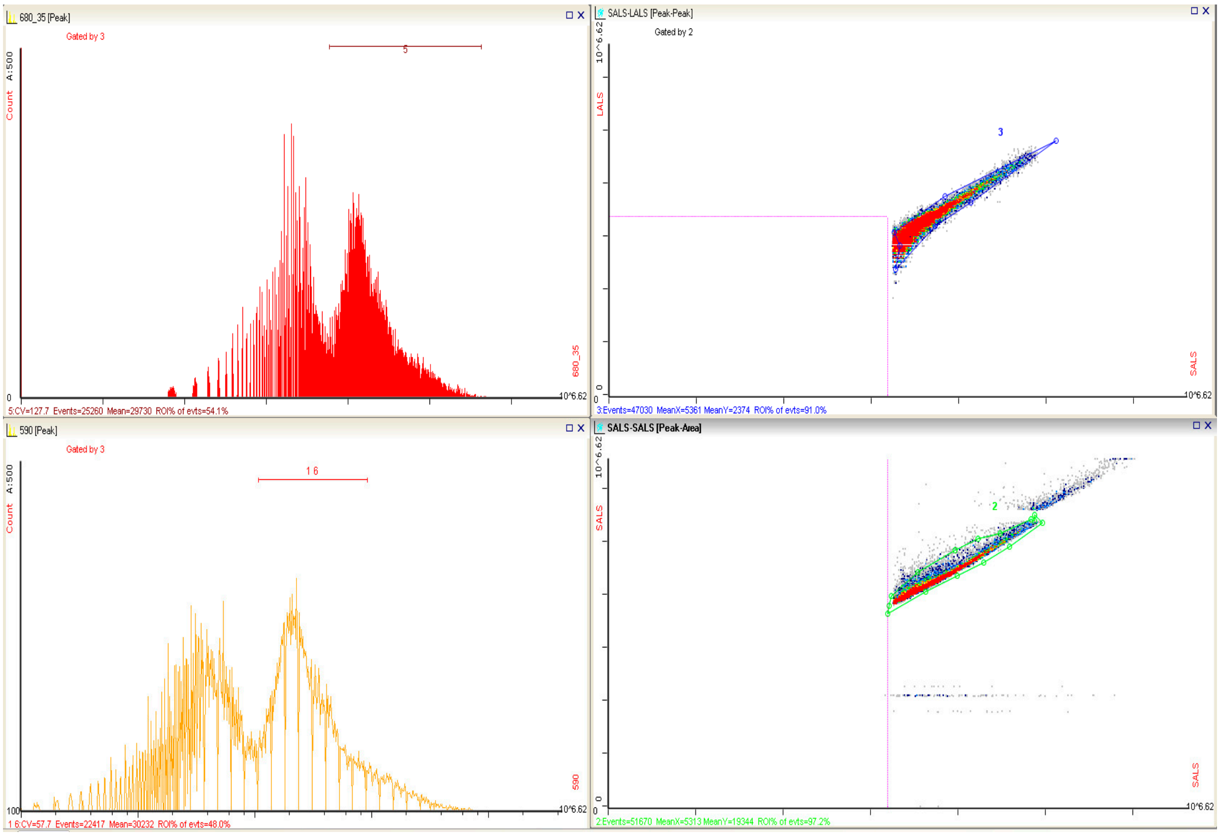

Figure S2. Viability of probiotics analyzed by flow cytometry – results of analysis of commercial probiotic mixture incubated in water medium.
